# Impact of mandatory indications for outpatient antibiotic orders on accurate tracking of antibiotic indications

**DOI:** 10.1017/ice.2024.88

**Published:** 2024-09

**Authors:** Charles Oertli, Milner Staub, Minhua Zhang, Sophie E. Katz

**Affiliations:** 1 Department of Medicine, Vanderbilt University Medical Center, Nashville, TN, USA; 2 Department of Pediatrics, Vanderbilt University Medical Center, Nashville, TN, USA; 3 Division of Infectious Diseases, Department of Medicine, Vanderbilt University Medical Center, Nashville, TN, USA; 4 Infectious Diseases Section, Medical Service Line, Veterans Affairs Tennessee Valley Healthcare System, Nashville, TN, USA; 5 Quality, Safety, and Risk Prevention, Vanderbilt University Medical Center, Nashville, TN, USA; 6 Division of Infectious Diseases, Department of Pediatrics, Vanderbilt University Medical Center, Nashville, TN, USA

## Abstract

**Objective:**

We sought to evaluate whether implementing mandatory indications for outpatient electronic antibiotic orders or using encounter International Classification of Diseases, Tenth Revision (ICD10) codes more accurately reflected clinicians’ charted diagnosis in encounter notes. Secondarily, we examined the appropriateness of antibiotic prescriptions.

**Design:**

Cross-sectional study.

**Methods:**

Mandatory indications were added to all outpatient electronic antibiotic orders on May 18, 2022. A randomly selected convenience sample of 1300 outpatient encounters with antibiotics from walk-in clinics was reviewed. Adjusted logistic regression was used to compare the congruence between encounter ICD10 code and charted diagnosis for encounters from July 15 to September 15, 2021 (pre-implementation period) to the congruence between encounter ICD10 code, charted diagnosis, and mandatory indication for encounters from July 15 to September 15, 2022 (post-implementation period). Antibiotic appropriateness based on charted diagnosis was also evaluated.

**Results:**

Among 1300 outpatient encounters, congruence between charted diagnosis and ICD10 code significantly increased in the post-implementation period (87.7% (565/644)) versus pre-implementation (83.3% (540/648), adjusted odds ratio (aOR) 1.52; 95% CI 1.03–2.25). Congruence between charted diagnosis and mandatory indication during post-implementation was 95.2% (613/644) and >5 times more likely to be congruent than charted diagnosis and ICD10 code during pre-implementation (aOR 5.45; 95% CI 3.26–9.11). Antibiotic prescribing based on charted diagnosis was twice as likely to be appropriate in the post-implementation period (aOR1.99; 95% CI 1.32–2.98).

**Conclusions:**

Mandatory indications within antibiotic orders show better congruence with charted diagnosis than ICD10 codes and may increase antibiotic appropriateness and congruence between ICD10 code and charted diagnosis.

## Background

Antibiotics are among the most common outpatient prescriptions in both adults and children with approximately 270 million outpatient antibiotic prescriptions written in the United States in 2016.^1–3^ Antimicrobial stewardship programs (ASPs) have been implemented at healthcare institutions worldwide to combat antibiotic overuse and misuse, which lead to increased antimicrobial resistance, unnecessary risk of medication side effects, and increased healthcare costs.^3–5^ A key principle of ASPs is monitoring current prescribing patterns to track data and find opportunities for improvement in antimicrobial use.^4,5^ The Centers for Disease Control and Prevention’s Core Elements of Outpatient Antibiotic Stewardship recommend that antimicrobial prescriptions include documentation of the dose, duration, and indication to aid tracking and feedback efforts.^3^

A common method to collect antimicrobial indications data is a point prevalence survey, in which all antimicrobial prescriptions over a specific period are examined and cross referenced via manual review for their charted diagnosis.^6^ This review process can be time-consuming and does not provide real-time data on prescribing patterns that are necessary to enact changes quickly.

Computerized Physician Order Entries (hereafter referred to as “electronic orders”) that reside within electronic medical records (EMRs) allow for quick and easy electronic order entry and have become a common means of prescribing antimicrobials.^5,7^ Electronic orders have the potential for quality monitoring of prescriptions in real time; and therefore, can assist ASPs in ensuring appropriate antimicrobial prescribing at a healthcare institution.^6,7^

Two potential methods to track indications for antimicrobial prescriptions without using manual chart review are utilizing *International Classification of Diseases, Tenth Revision* (ICD10) codes linked to encounters with antimicrobial prescriptions, or including an indication function for each antimicrobial electronic order.^6–8^ In the inpatient setting, there is evidence of a high, yet variable, congruence (74%–100%) between mandatory antibiotic indication functions and the charted diagnosis.^8–11^ Accuracy of ICD10 codes or mandatory indications for intent of tracking antibiotic prescription indications in the outpatient setting is largely unknown; congruence between provider-selected indications and charted diagnosis has been estimated at 92.5%.^12^ Walk-in clinics, as part of the urgent care system, present a unique setting with a high patient turnover rate, lack of patient continuity, and a high percentage – 20%–40% on average^13^ – of encounters resulting in an antibiotic prescription.

After implementing mandatory, provider-selected, antibiotic indications in the outpatient setting across our healthcare system, our primary aim was to examine to what extent mandatory indications accurately reflected charted diagnosis in the corresponding encounter note for outpatient walk-in clinic encounters. Secondary aims were to examine the degree of congruence between mandatory indications and charted diagnosis (post-intervention) compared to provider-selected encounter ICD10 codes and charted diagnosis (pre-intervention). Additionally, we assessed appropriateness of antibiotic prescriptions for the charted diagnosis.

## Methods

### Walk-in-clinics (WICs)

Our WICs are urgent care clinics staffed by a mix of family medicine and internal medicine physicians, nurse practitioners, and physician assistants treating adult patients and pediatric patients >2 months of age. These are not retail clinics nor acute care clinics associated with primary care offices. Our WICs are open Monday to Friday from 7:30 a.m. to 7:30 p.m. and weekends from 8 a.m. to 5 p.m. The WICs are located throughout Middle Tennessee. Over 219,000 patient encounters occur annually in the WICs, 77.5% (170,458/219,838) are adult patient encounters and 60.5% (132,936/219,838) are for patients who identify as female. Most patients, 76.6% (168,444/219,838) are white, 9% (19,789 /219,838) are black, and 6.8% (14,911/219,838) are Hispanic/Latino. Nurse practitioners see 51.6% (113,408/219,838) of encounters while physicians see 31.2% (68,621/219,838), and physician assistants see 17.1% (37636/219,838) of encounters. There is access to rapid streptococcal, COVID-19, and influenza testing as well as full laboratory services and X-ray services. The types of conditions typically treated in our WICs are upper respiratory tract infections, sexually transmitted infections, rashes, headaches, diarrhea, and other minor injuries and acute illnesses.

### Intervention

Mandatory indication functions were introduced into antibiotic electronic orders within our commercially available EMR (Epic) on May 18, 2022 (Supplemental Figure 1). These indications were chosen based on analysis of the most frequently used ICD10 codes associated with outpatient encounters in which an antibiotic was prescribed. Prior to implementation, adult and pediatric clinical practice committees approved the selected indication choices and offered institutional support for this intervention. No specific individual clinician education was provided; although, the institution had previously implemented inpatient antibiotic mandatory indications. Notably, WIC providers typically do not practice inpatient services. Despite this lack of exposure, no technical or cultural barriers were encountered.

### Study design

We performed a cross-sectional review of all outpatient adult and pediatric encounters with antibiotics prescribed at 12 Vanderbilt University Medical Center walk-in clinics (WICs) from July 15 to September 15, 2021 (pre-implementation period), compared to July 15 to September 15, 2022 (post-implementation period). We selected a convenience sample of 650 encounters from both pre- and post-implementation periods for chart review and analysis using Microsoft Excel’s random number generator function to select row numbers from a list of all prescribed antibiotics from face-to-face encounters in these periods. For encounters with multiple antibiotic prescriptions, each antibiotic was listed as a separate row. We compared the ICD10 codes to the charted diagnosis based on review of the assessment and plan section of the provider’s encounter note. If the ICD10 codes affiliated with the encounter were not congruent with the charted diagnosis written in their note, this ICD10 code-charted diagnosis pair was labeled “incongruent.” If the charted diagnosis matched any one of the ICD10 codes, the ICD10 code-charted diagnosis pair was labeled “congruent.” If the mandatory indication matched the charted diagnosis, this mandatory indication-charted diagnosis pair was labeled “congruent.” If the mandatory indication did not match the charted diagnosis, this mandatory indication-charted diagnosis pair was labeled “incongruent.” Congruence between ICD10 code and mandatory indication was not analyzed. The gold standard for intent of the antibiotic prescription was the charted diagnosis (rather than ICD10 code or mandatory indication). For encounters with multiple antibiotics prescribed, only the antibiotic that was assigned for review during randomization was included in statistical analysis. We did not look at previous encounters to see if a patient had been prescribed another antibiotic prior to the encounter during the study period.

### Appropriateness of antibiotic prescriptions

WICs have a document that outlines empiric antibiotic recommendations based on diagnosis developed by a WIC provider and reviewed by our pediatric and adult ASPs which incorporates national guidelines and local antibiogram data. We assessed appropriateness of antibiotic prescriptions, classified as necessary, effective, and optimal choice, by comparing the charted diagnosis for the antibiotic with these guidelines. (Supplemental Table 2). Each antibiotic and charted diagnosis pair were independently reviewed for appropriateness and documented as appropriate, inappropriate based on local guidelines (but appropriate based on national or societal guidelines), or inappropriate based on national or local guidelines (hereafter referred to as “inappropriate”) by two infectious diseases physicians (MS and SK). Discordant assessments were flagged, and consensus was reached through discussion. Only appropriateness of antibiotic choice, not antibiotic dose or duration, was assessed.

### Statistical analysis

We calculated percent congruence for ICD10 codes and charted diagnosis in both pre- and post-indication groups. We calculated percent congruence between mandatory indication and charted diagnosis in the post-implementation group. We performed multivariable logistic regression to determine adjusted odds ratios (aOR) and 95% confidence intervals (95% CI) for both antibiotic congruence and appropriateness in the pre- and post-implementation periods, adjusting for antibiotic choice and charted diagnosis, clustered by prescriber. For the logistic regression outcome of appropriateness, we created a binomial variable of appropriate or inappropriate. Prescriptions in the “appropriate” variable were those categorized as appropriate by either local or national guidelines. This method was chosen to give the most conservative estimate by giving the benefit of the doubt to providers who may have used more global resources to choose which antibiotic to prescribe instead of the local guidance documents. For each diagnosis, we performed a two-sample T-test to evaluate the difference in congruence between the charted diagnosis and ICD10 code in the pre-implementation group and between the charted diagnosis and selected mandatory indication in the post-implementation group. We also performed a Pearson Chi Square test to evaluate the change in encounters classified as appropriate, inappropriate based on local guidelines, and inappropriate between pre- and post-intervention periods. All analyses were done using STATA (STATA/MP 16.1, College Station, Texas).

## Results

We reviewed 650/10112 (6.4 pre-implementation and 650/10562 (6.2%) post-implementation encounters. We excluded two pre-implementation encounters for insufficient written documentation and 6 post-implementation encounters (two for insufficient written documentation and four for bypassing the electronic order mandatory indication function resulting in no indication on the antibiotic prescription).

### Congruence

We found that 83.3% (540/648) of pre-implementation and 87.7% (565/644) of post-implementation encounters had congruent antibiotic indication based on charted diagnosis and associated ICD10 code for the encounter. Congruence between charted diagnosis and mandatory indication in the post-implementation period was 95.2% (613/644) (Table 1). Post-implementation congruence (between mandatory indication and charted diagnosis) was 5.4 times more likely than pre-implementation congruence (between ICD10 code and charted diagnosis) (aOR 5.4; 95% CI 3.3–9.1).

**Table 1.** Pre- and post-implementation encounter characteristics

	Pre-indication encounters (N=648)	Post-indication encounters (N=645)
Providers	100	91
Antibiotic		
Amoxicillin	208 (32.1%)	182 (28.2%)
Amoxicillin/clavulanate	102 (15.7%)	123 (19.1%)
Cefdinir	61 (9.4%)	47 (7.3%)
Azithromycin	58 (9.0%)	57 (8.8%)
Cephalexin	51 (7.9%)	54 (8.4%)
Doxycycline	47 (7.3%)	63 (9.8%)
Nitrofurantoin	36 (5.6%)	31 (4.8%)
Sulfamethoxazole/ Trimethoprim	30 (4.6%)	31 (4.8%)
Clindamycin	17 (2.6%)	16 (2.5%)
Penicillin	13 (2.0%)	11 (1.7%)
Metronidazole	11 (1.7%)	3 (0.5%)
Ciprofloxacin	9 (1.4%)	17 (2.6%)
Levofloxacin	4 (0.6%)	5 (0.8%)
Cefuroxime	1 (0.2%)	2 (0.3%)
Clarithromycin	0 (0.0%)	2 (0.3%)
Fosfomycin	0 (0.0%)	1 (0.2%)
Charted diagnosis		
Group A streptococcus	221 (34.1%)	196 (30.4%)
Skin/soft tissue infection	81 (12.5%)	94 (14.6%)
Sinusitis	83 (12.8%)	86 (13.3%)
Urinary tract infection	76 (11.7%)	91 (14.1%)
Acute otitis media	74 (11.4%)	76 (11.8%)
Pneumonia	27 (4.2%)	19 (3.0%)
Bronchitis	26 (4.0%)	18 (2.8%)
Prophylaxis	21 (3.2%)	14 (2.2%)
Other	39 (6.0%)	51 (7.9%)
Congruence charted diagnosis vs ICD10 code	540 (83.3%)	566 (87.8%)
Congruence charted diagnosis vs mandatory indication		614 (95.2%)

Percentage reflects the percentage of total encounters within pre- or post-implementation groups, respectively. Charter diagnosis of “Other” includes Intestinal infection, Viral Upper Respiratory Tract Infection, Patient Request, Histoplasmosis, Septic Joint, Chronic Obstructive Pulmonary Disease Exacerbation, Bacterial Vaginosis, Tonsillitis and Pharyngitis Not Otherwise Specified, Dental Infection, Sexually Transmitted Infections, Tickborne Illness, Appendicitis, Prostatitis, COVID, Asthma, and Epididymitis.

### Analysis by charted diagnosis

**Figure 1.**
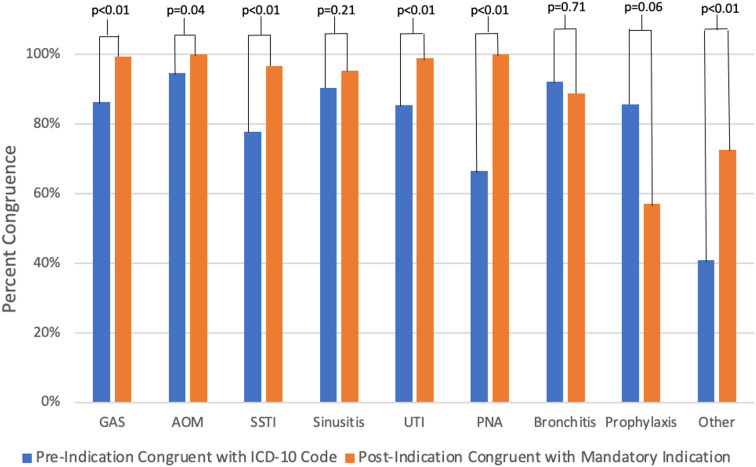
Congruent Encounters Based on Charted Diagnosis, International Classification of Diseases, Tenth Revision (ICD10) Code, and Mandatory Indication. Congruence of charted diagnosis to ICD10 and mandatory indication among pre- and post-implementation groups. Congruence in pre-implementation group reflects congruence between ICD10 code and charted diagnosis. Congruence in the post-implementation group reflects congruence between ICD10 code and mandatory antibiotic electronic order indication. GAS, group A streptococcus pharyngitis, AOM, acute otitis media, SSTI, skin and soft tissue infection, UTI, urinary tract infection, PNA, pneumonia. Other = diagnoses with <10 encounters in the pre-implementation group.

Streptococcal pharyngitis (GAS) was the most frequent charted diagnosis in both pre- and post-indication encounters (34.1% (221/648) pre vs 30.4% (196/645) post). When comparing congruence in pre-implementation charted diagnosis and ICD10 code and post-implementation charted diagnosis and mandatory indication, every charted diagnosis category increased in percent congruence except for bronchitis (92.3% pre vs 88.9% post, difference –3.4%; *P* = .71) and prophylaxis (85.7% pre vs 57.1% post, difference –28.6%; *P* = .06). Among encounters in which ICD10 code and charted diagnoses were incongruent, the most common charted diagnoses in both the pre- and post-implementation groups were streptococcal pharyngitis (GAS), skin and soft tissue infection (SSTI), urinary tract infection (UTI), and Other (Figure 1). Among encounters in which mandatory indication and charted diagnoses were incongruent in the post-implementation group, the most common charted diagnoses were Other (14/31; 45%), Prophylaxis (6/31; 19%), and Sinusitis (4/31, 13%) (Figure 1).

### Appropriateness based on charted diagnosis

**Figure 2.**
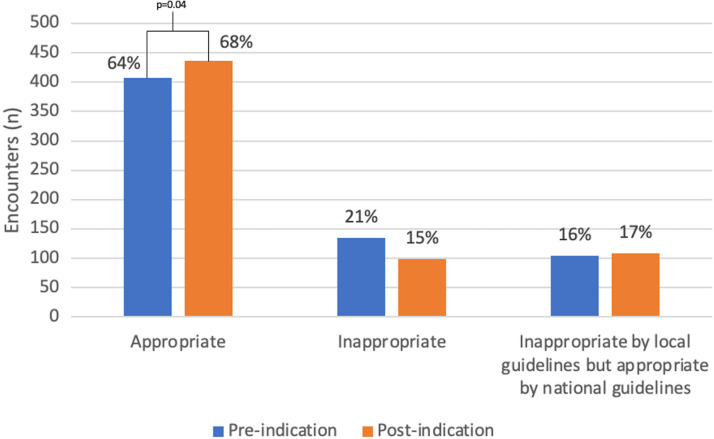
Appropriateness of Antibiotic Prescriptions. Pre- and post-implementation appropriateness with inappropriate encounters stratified by reason for inappropriateness (either nonadherence to national guidelines or nonadherence to local guidelines). Antibiotics were significantly more likely to be appropriate (*X* ^2^ = 6.6, *P* = .04) in the post-implementation period. Percentage reflects percentage of total encounters within pre- or post-implementation groups, respectively.

Among pre-implementation encounters, 63% (408/648) were appropriate for the charted diagnosis, 20.8% (135/648) were inappropriate, and 16.2% (105/648) were inappropriate based on local guidelines but appropriate based on national guidelines (Figure 2). In the post-implementation period, appropriateness increased to 68% (436/644), 15% (99/644) were inappropriate due to nonadherence to national guidelines, and 17% (109/644) were inappropriate based on local guidelines but appropriate based on national guidelines (Figure 2).

In the post-implementation period, antibiotics were significantly more likely to be appropriate (aOR1.99; 95% CI 1.32–2.98). Excluding encounters that were inappropriate based on local guidelines but appropriate based on national guidelines and comparing appropriate to inappropriate based on national guidelines, prescriptions written in post-implementation encounters remained more likely to be appropriate (aOR1.90; 95% CI 1.12–3.21). There was a significant change in the number of appropriate, inappropriate based on local guidelines but appropriate based on national guidelines, and inappropriate prescriptions between pre- and post-intervention periods (*P* = .04).

## Discussion

Mandatory indications were more reliable than ICD10 codes for accurately tracking outpatient antibiotic prescription indications, with 95.2% congruence between charted diagnosis and mandatory indication in the post-implementation period, compared to 83.3% between charted diagnosis and ICD10 code in the pre-implementation period and 87.7% between charted diagnosis and ICD10 code in the post-implementation period. These data align with Papanikolla, *et al* findings which reported 92.5% congruence between outpatient mandatory indications and charted diagnoses.^12^ Encounter diagnoses showed increased congruence with mandatory indication functions when compared to ICD10 codes, with the exceptions of antibiotic prescriptions for bronchitis and prophylaxis. Bronchitis may have had decreased congruence with mandatory indication due to the addition of the phrase “(antibiotics typically not indicated)” when providers selected bronchitis as their mandatory indication for the desired antibiotic (Supplemental Figure 1). Prophylaxis is a broad (Pneumocystis jirovecii, recurrent UTI, tick/animal bites, etc), though rarely selected, indication for antibiotics. It is unclear why congruence for prophylaxis decreased in the post-implementation group, but it may be due to providers selecting similar indications (ie selecting SSTI when charted diagnosis was prophylaxis for dog bite). Future analysis using a larger sample size and focused analysis on encounters with antibiotic prophylaxis as the selected indication may elucidate the apparent countertrend in the prophylaxis indication. The diagnoses with greatest increase in congruence after implementation of mandatory indications were the most common overall diagnoses; GAS, SSTI, and UTI.

Surprisingly, both antibiotic appropriateness and congruence between ICD10 code and charted diagnoses increased in the post-implementation group compared to the pre-implementation group. This may be explained by the concept of *accountable justification* where providers are asked to document a justification for each prescription and are therefore more likely to preserve their professional reputation by acting in line with injunctive norms and recommended clinical guidelines.^14–16^ Although not directly asked to justify the antibiotic choice and indication, by having to choose the associated indication, the provider, is in effect, having to justify that prescription.

Despite the effect of accountable justification, we found a high percent of inappropriate antibiotic choice in both the pre- and post-implementation groups (37% and 32%, respectively). These findings were consistent with previously published literature where 14.4%–49% of outpatient antibiotic prescriptions were inappropriate.^13,17–19^ However, implementation of mandatory indications was associated with a significant decrease in the rate of inappropriate antibiotic prescribing in the post-implementation group. The main reason for inappropriate prescriptions was due to a high degree of failure to follow both national guidelines and local guidance. It is particularly challenging to counter national guidelines when they may not provide the best treatment based on local antibiograms, despite existence of local guidance documents, due to the ready availability of national resources. For example, at our institution, cephalexin has good susceptibility against *Escherichia coli* and *Klebsiella pneumoniae*, has good bioavailability, and gets adequate penetration into the urine to effectively treat cystitis, making cephalexin a good choice for simple cystitis. However, national guidelines list cephalexin as a less well-studied choice compared to nitrofurantoin, trimethoprim-sulfamethoxazole, or fosfomycin due to lack of data.^20^ Notably, UTI guidelines are pending a new update and there has been subsequent literature to support use of cephalexin for uncomplicated UTI.^20–23^ Dissemination of updated evidence and implementation into practice is often delayed.^24,25^

This study had limitations. Four of 650 (0.62%) encounters were excluded from the post-implementation group for bypassing the mandatory antibiotic electronic order indication function due to our EMR upgrade, which implemented the mandatory indication function, but did not alter providers’ previously preset “favorite” orders; however, this represents a small portion of all encounters and would not likely skew the data. Future EMR upgrades and implementation plans should include provisions to update providers’ favorite orders as well. Additionally, because this study was only performed at WICs affiliated with a single, tertiary care academic center in Middle Tennessee, it may not be generalizable to other settings or populations; however, our findings are similar to those published at another institution, which suggests this may be a shared finding.^12^ We picked a convenience sample size of 650 encounters in both the pre- and post-implementation groups, which may have led to unintentionally skewed data; however, we randomized charts prior to selecting those to review, and this is the largest study of its kind to date with 1300 total encounters included, decreasing the likelihood that we may have missed a significant trend. We did not survey providers to assess their acceptance of mandatory antibiotic indications and their impact on workflow, which will be topics for future research. When ascertaining antibiotic appropriateness, investigators were not blinded to pre- or post-implementation groups, presenting another potential source of bias; however, clear criteria for appropriateness were outlined and documented based on the charted diagnosis and antibiotic choice combination, which was applied universally. This measure likely limited the degree of impact of subjective opinion on determining appropriateness. Finally, appropriateness may have increased secondary to other antimicrobial stewardship efforts made during this time; however, we were unable to effectively capture and measure this effect.

Despite these limitations, our study supports use of mandatory antibiotic electronic order indications as a reliable method to accurately assess antibiotic prescriptions for outpatient ASPs. Implementation of mandatory antibiotic electronic order indications allows ASPs to monitor prescribing patterns in real time and provide reliable and valid individual antibiotic prescribing feedback to providers. More data are needed on end-user perception of mandatory indications and on generalizability of this method in different regions and clinical settings.

## Supporting information

Oertli et al. supplementary material 1 Oertli et al. supplementary material

Oertli et al. supplementary material 2 Oertli et al. supplementary material

## Supplementary material

The supplementary material for this article can be found at https://doi.org/10.1017/ice.2024.88.
