# Supplementary material for: Impact of mandatory indications for outpatient antibiotic orders on accurate tracking of antibiotic indications
Source: Infect Control Hosp Epidemiol. 2024 May 13;45(9):1115–20. doi: 10.1017/ice.2024.88 (PMC11518663; doi:10.1017/ice.2024.88)
Supplement: Oertli et al. supplementary material 1 — Oertli et al. supplementary material [file S0899823X24000886sup001.docx]

**Supplemental Materials**

| **Age** | ≤18 years old: 49,380 (22.5%) | >18 years old: 170,458 (77.5%) |  |
| --- | --- | --- | --- |
| **Sex** | Female: 132,936 (60.5%) | Male: 86898 (39.5%) | Unknown: 7 (<0.01%) |
| **Race** | White: 168,444 (76.6%) | Black: 19,789 (9%) | Other: 31,605 (14.4%) |
| **Ethnicity** | Hispanic/Latino: 14,911 (6.8%) | Not Hispanic/Latino: 204,927 (93.2%) |  |
| **Primary Spoken Language** | English: 212,987 (96.9%) | Spanish: 4,405 (2%) |  |
| **Provider Type** | NP: 113,408 (51.6%) | Physician: 68,621 (31.2%) | PA: 37,636 (17.1%) |

**Supplemental Table 1. Demographic Data of Vanderbilt WIC encounters from 2023.** 219,838 total WIC encounters. N= number of encounters meeting specified criteria. (%) reflect percentage of total WIC encounters.

| **Charted Diagnosis** | **Appropriate** | **Inappropriate due to Local Guidance** | **Overly Broad or Guideline Inappropriate** |
| --- | --- | --- | --- |
| **Bronchitis or viral upper respiratory tract infection** | No antibiotic | No antibiotic | Any antibiotic |
| **UTI** | Cephalexin, amoxicillin-clavulanate, nitrofurantoin | Trimethoprim-sulfamethoxazole, cefdinir | Fluroquinolones, doxycycline |
| **SSTI** | Cephalexin, penicillin, amoxicillin, amoxicillin-clavulanate, trimethoprim-sulfamethoxazole | Doxycycline, Clindamycin (if adult), erythromycin | Fluroquinolones, cefdinir |
| **AOM** | Amoxicillin, amoxicillin-clavulanate | Cefdinir | Fluroquinolones, azithromycin, doxycycline, trimethoprim-sulfamethoxazole, cephalexin |
| **GAS Pharyngitis** | Amoxicillin, penicillin | Cephalexin, clindamycin, azithromycin | Cefdinir, fluroquinolones, amoxicillin-clavulanate |
| **Sinusitis** | Amoxicillin, amoxicillin-clavulanate, penicillin, doxycycline | Cefdinir, azithromycin | Trimethoprim-sulfamethoxazole, levofloxacin |
| **Pneumonia** | Amoxicillin, amoxicillin-clavulanate, penicillin, doxycycline, cefpodoxime, azithromycin, levofloxacin | Cefdinir | ciprofloxacin |

**Supplemental Table 2. Definitions of appropriateness**. Antibiotic prescription appropriateness classification (appropriate, inappropriate based on local guidelines, or inappropriate) based on charted diagnosis for antibiotic. UTI= urinary tract infection. AOM= acute otitis media. GAS= group A Streptococcus.

**Supplemental Figure 1. Antibiotic Computerized Order Entry Mandatory Indication Options.** Indication options that providers were forced to select within the electronic orders. Prescription could not be processed if an indication was not selected. In the above example no indication is selected so there is a red error message to the left of “indication” indicating that the order is not ready to be processed.
