# Supplementary figures and images for: Impact of mandatory indications for outpatient antibiotic orders on accurate tracking of antibiotic indications
Source: Infect Control Hosp Epidemiol. 2024 May 13;45(9):1115–20. doi: 10.1017/ice.2024.88 (PMC11518663; doi:10.1017/ice.2024.88)

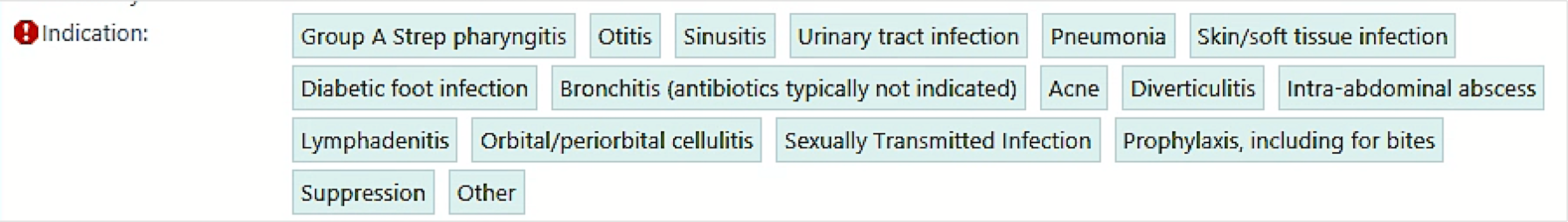

Supplement: Oertli et al. supplementary material 2 — Oertli et al. supplementary material [file S0899823X24000886sup002.tiff]
